# Severity of Depressive Symptoms by Perceived Stress and Gut Microbiota Composition: A Sex‐Stratified Bayesian Approach in a Non‐Clinical Sample

**DOI:** 10.1002/smi.70152

**Published:** 2026-02-17

**Authors:** Sylvia De Napoli, Tania Moretta, Sebastiano Ravenda, Leonardo Mancabelli, Francesca Turroni, Andrea Sgoifo, Marco Ventura, Luca Carnevali

**Affiliations:** ^1^ Stress Physiology Lab Department of Chemistry Life Sciences and Environmental Sustainability University of Parma Parma Italy; ^2^ Institute of Cognitive Sciences and Technologies National Research Council of Italy Rome Italy; ^3^ Department of Theoretical and Applied Sciences eCampus University Como Italy; ^4^ Department of Medicine and Surgery University of Parma Parma Italy; ^5^ Microbiome Research Hub University of Parma Parma Italy; ^6^ Laboratory of Probiogenomics Department of Chemistry Life Sciences and Environmental Sustainability University of Parma Parma Italy

**Keywords:** depression, *Eubacterium*, gut microbiota, sex differences, stress

## Abstract

Gut dysbiosis, an imbalance in gut microbiota, is increasingly linked to depression through the microbiota‐gut‐brain axis. Stress is an important risk factor for both gut dysbiosis and depression. Despite evidence of altered gut microbiota composition in patients with depression, little is known about how stress and specific gut microbiota features interact to influence depressive symptoms in healthy individuals. This study examined 398 healthy adults (241 women) who provided stool samples and completed validated questionnaires on perceived stress (PSS) and depressive symptoms (CES‐D), with a focus on sex differences. In this sample, men and women were characterised by similar gut microbiota composition and diversity. Women reported higher PSS scores than men, whereas no differences were found in CES‐D scores. Using Bayesian analyses, results showed that perceived stress predicted depressive symptoms in both sexes. Notably, in women, the genus *Eubacterium* moderated this relationship: higher perceived stress combined with lower *Eubacterium* abundance predicted more severe depressive symptoms. In contrast, no moderations by gut bacteria were found in men. The current results warrant further sex‐specific investigations of the interaction between stress and specific gut microbiota features in influencing depressive symptoms and suggest that the genus *Eubacterium* might be a promising microbial biomarker associated with depressive symptoms, particularly in women under higher stress levels.

## 1 Introduction

The microbiota‐gut‐brain (MGB) axis, which integrates immune, neuroendocrine, and vagal pathways, has been suggested to play a key role in the development and progression of stress‐related disorders, including depression (Cryan and Dinan 2012; Dinan and Cryan 2017). Supporting its involvement, several studies report that patients with depression are often affected by gut dysbiosis (reviewed in Sanada et al. 2020). This imbalance can trigger inflammation and autonomic changes, alter the production of microbial derived metabolites like short‐chain fatty acids (SCFAs), disrupt neurotransmitter levels, and affect epigenetic processes, all contributing to depressive symptoms (Cryan and Dinan 2012; Dinan and Cryan 2017). As such, attempts have been made to identify specific gut microbial taxa involved in the imbalance of gut microbiota composition that characterises depression. For example, several studies indicate that patients with depression, some of which exhibit comorbid gastrointestinal disorders, present a lower abundance of gut bacteria belonging to the *Faecalibacterium*, *Coprococcus*, and *Eubacterium* genera (Sanada et al. 2020; Yang et al. 2020). These bacteria genera are known to produce SCFAs, which have emerged as peculiar mediators of microbiota‐gut‐brain interactions due to their capacity to influence intestinal barrier integrity, immune response, and afferent vagal pathways (Dalile et al. 2019; Lal et al. 2001; Lin et al. 2024). Relatedly, the important role of the vagus nerve in microbiota‐gut‐brain signalling in mood disorders (Bonaz et al. 2018; Chang et al. 2024; Faraji et al. 2025) is emphasised by findings that the beneficial effects of probiotics on both gut microbiota composition and depressive‐like symptoms are abolished following vagotomy in animal models (Bravo et al. 2011). Additionally, a recent study demonstrated that low‐vagally mediated heart rate variability, a surrogate measure of cardiac vagal activity, is associated with greater depressive symptoms and altered gut microbiota composition in a small sample of healthy individuals (Ravenda et al. 2025).

Similarly, patients with gastrointestinal disorders, such as inflammatory bowel disease (IBD), frequently experience mood and neuropsychiatric disorders, which may be due to disrupted communication between the gut microbiota and the brain (Petracco et al. 2025). For instance, a lower gut taxonomic diversity and a decreased abundance of beneficial SCFA‐producing bacteria, such as those belonging to the *Faecalibacterium* and *Roseburia* genera, were found to characterise the gut microbial composition of patients with IBD (Gevers et al. 2014), and particularly those with comorbid depression (D.‐L. Chen et al. 2021).

The central role of the MGB axis in the comorbidity between mood and gastrointestinal disorders is also supported by a growing body of preclinical and clinical research showing that modulation of the gut microbiota can aggravate or ameliorate both behavioural and gastrointestinal symptoms (Kelly et al. 2016; Sarkar et al. 2016; Zheng et al. 2016). Likewise, therapeutic interventions targeting behavioural symptoms have been shown to influence gut microbiota composition (Wang et al. 2022), further reinforcing the bidirectional nature of communication between gut bacteria and the brain.

Importantly, stressful life events represent a well‐known risk factor for the development of depression, and both stress and depression are often associated with autonomic and immune dysfunctions (Carnevali et al. 2018; Kendler et al. 1999; H.‐G. Kim et al. 2018; Sgoifo et al. 2015). It has also been demonstrated that stress affects gastrointestinal functionality, for example by increasing intestinal permeability (Morys et al. 2024), which can lead to several changes in gut microbiota composition in both humans and animal models (Bharwani et al. 2016; Foster et al. 2017; Leigh et al. 2023; Molina‐Torres et al. 2019).

However, while most investigations focus on the study of gut microbiota composition in patients with depression, it is still unknown whether the interaction between stress and specific gut microbiota features is associated with depressive symptomatology severity in healthy individuals. Such an investigation would provide important insights into the interplay between gut microbiota composition, depressive symptoms, and stress in non‐clinical populations. Moreover, the higher prevalence of depression among women compared to men (S. Li et al. 2023) and the different changes in gut microbiota composition found between male and female patients with depression (Niemela et al. 2024) suggest considering biological sex in these associations.

Therefore, the primary objective of the current study was to investigate the extent to which perceived stress and specific features of gut microbiota composition are associated, independently and/or in combination, with the severity of depressive symptoms in a large sample of healthy individuals, with a focus on sex differences.

## 2 Methods

### 2.1 Participants

The present study is based on data collected from healthy adults recruited within the framework of the Parma Microbiota project (Mancabelli et al. 2024). The project was approved by the local ethics Committee (Comitato Etico dell’Area Vasta Emilia Nord, Emilia‐Romagna Region, Italy, under the ID 1107/2020/TESS/UNIPR) and all procedures were performed in accordance with the Declaration of Helsinki.

To be eligible for inclusion in the Parma Microbiota project, participants were required to be resident in the province of Parma (Italy) and to be at least 18 years old. Exclusion criteria included the presence, at the time of the assessment, of gastrointestinal symptoms/signs (diarrhoea, abdominal pain, constipation, etc.) or systemic symptoms (low grade fever, arthralgia) suggestive of the presence of IBD or other unknown acute or chronic gastroenterological pathologies, and use of antibiotic therapies during the 20 days prior to the laboratory assessment. Further exclusion criteria for the present investigation included past or present use of psychoactive drugs or diagnosis of a psychiatric or mood disorder.

A final sample of 398 individuals (241 women), ranging in age from 18 to 85 years (*M* = 49.6, SD = 15.3), was included in the present investigation. After signing the consent form, participants completed a series of socio‐demographic and dispositional scales (see below “Psychometric questionnaires”). Subsequently, they were instructed orally and were also given written instructions on how to collect, store, and deliver one stool sample on the next day.

### 2.2 Analysis of the Microbiota Composition

The metagenomic data used in this study were part of the Parma Microbiota project (Mancabelli et al. 2024) and are publicly available as shotgun metagenomic datasets under the accession number PRJNA1046438. To assess the microbiota composition at the genus level, the subsample of 398 healthy subjects was reanalysed through the METAnnotatorX2 software following the standard filtering parameters reported in the manual (Mancabelli et al. 2024; Milani et al. 2018). In detail, the fastq files were filtered to remove reads with a quality of < 25 and to retain reads with a length of > 100 bp. Subsequently, human host DNA filtering was performed through Bowtie 2 software (Langmead et al. 2019; Langmead and Salzberg 2012), following the METAnnotatorX2 manual (Milani et al. 2021). Afterwards, the taxonomic classification of 100,000 reads was achieved by means of MegaBLAST (Y. Chen et al. 2015) employing a manually curated and pre‐processed database of genomes retrieved from the National Centre for Biotechnology Information, following the METAnnotatorX2 manual (Milani et al. 2021). We considered the relative abundance of gut bacteria at the genus level and indices of microbiota gut diversity, including Genus Richness and Shannon and Simpson indices, and measures to assess gut microbiota composition similarities between the groups (Principal Coordinate Analysis, PCoA).

### 2.3 Psychometric Questionnaires

The Perceived Stress Scale (Cohen et al. 1983) was used as a self‐report to measure the subjective perception of stress over the last month. Specifically, the PSS evaluates the degree to which a set of situations are perceived as stressful. It includes six items, each rated on a five‐point scale ranging from “Never (0)” to “Very often (4)”. A score between 14 and 26 reflects moderate perceived stress and a score equal to 27 or above reflects high perceived stress. In our sample, the PSS Cronbach's alpha was excellent (*α* = 0.86).

To assess depressive symptomatology during the past week, participants were asked to complete the Centre for Epidemiological Studies Depression Scale (CES‐D). The CES‐D is a common tool to assess the frequency of depressive symptoms in the general population (Radloff 1977). It includes 20 items, each rated on a 4‐point scale ranging from 0 (not at all) to 3 (a lot). Standard cutoffs are > 16 for mild depression and > 23 for clinical depression. In our sample, the CES‐D Cronbach's alpha was excellent (*α* = 0.91).

### 2.4 Statistical Analyses

All statistical analyses were performed using R software (R Development Core Team, 2016). Statistical significance set at *p* < 0.05. The normal distribution of variables was evaluated using the Shapiro‐Wilk Normality test and the Theoretical quantile (Q‐Q plot) test. Psychometric scores were normally distributed; thus, sex differences were assessed using Student's *t*‐tests. The relative abundances of bacteria genera were non‐normally distributed; thus, sex differences were evaluated using the Mann‐Whitney *U* test. Alpha and beta diversity analyses were conducted using the “vegan” package. The Shannon Index and Richness were normally distributed, with sex differences assessed via Student's *t*‐tests. The Simpson Index was non‐normally distributed; thus, sex differences were evaluated using the Wilcoxon test. Similarities between women and men gut microbiota composition (beta diversity) were assessed via PCoA calculated with the Bray‐Curtis dissimilarity matrix. Permutation analysis was conducted with Adonis under a reduced model to estimate possible significant sex differences.

Bayesian adaptive sampling for variable selection and model averaging was used to assess the existence of associations between psychological stress (i.e., PSS score) and gut bacteria genera, as well as their two‐way interactions (i.e., statistical predictors), with depressive symptoms (i.e., CES‐D score – dependent variable). Analyses considered only bacteria genera with a relative abundance > 1%, and were conducted separately for women and men taking age into account. The present Bayesian approach was used to assess what combination of statistical predictors provided an adequate description of the distributions that generated the observed depressive symptoms (R package: BAS; Clyde 2022). As an extension of Bayesian inference, this approach accounts for parameter uncertainty, through prior distributions, and model uncertainty, deriving posterior distributions for both the parameters and the models via Bayes' theorem, allowing for model selection and combined estimation (Armagan et al. 2011; Fragoso et al. 2016). Moreover, this approach accounts for multiple testing by considering all predictors simultaneously and penalising model complexity. Therefore, no additional correction for multiple comparisons was applied. Models were estimated by a Markov Chain Monte Carlo (MCMC) sampling method by using the Zellner–Siow Cauchy prior on the coefficients and a uniform prior distribution over the models.

Multicollinearity was monitored by examining the variance inflation factor (VIF). Before running the Bayesian analyses, all variables were centred and scaled. The null hypothesis was rejected when the 95% Bayesian credibility intervals (BCIs) did not include the null value (Kruschke 2015). Effect sizes are reported as posterior mean regression coefficients (*β*) and their associated 95% BCIs.

## 3 Results

### 3.1 Participants Characteristics

A total of 398 individuals were included in the present study, including 241 women and 157 men. There were no discrepancies between sex assigned at birth and self‐reported gender among the participants. The average age (mean ± SD) was similar between women (49.6 ± 14.5 years) and men (49.0 ± 16.4 years) (*p* = 0.7). Body mass index (BMI) was significantly different between the groups, with women having a lower average BMI (22.9 ± 3.6 kg/m^2^) compared to men (24.9 ± 3.9 kg/m^2^; *p* < 0.001).

### 3.2 Characterisation of the Gut Microbiota Composition

**FIGURE 1:**
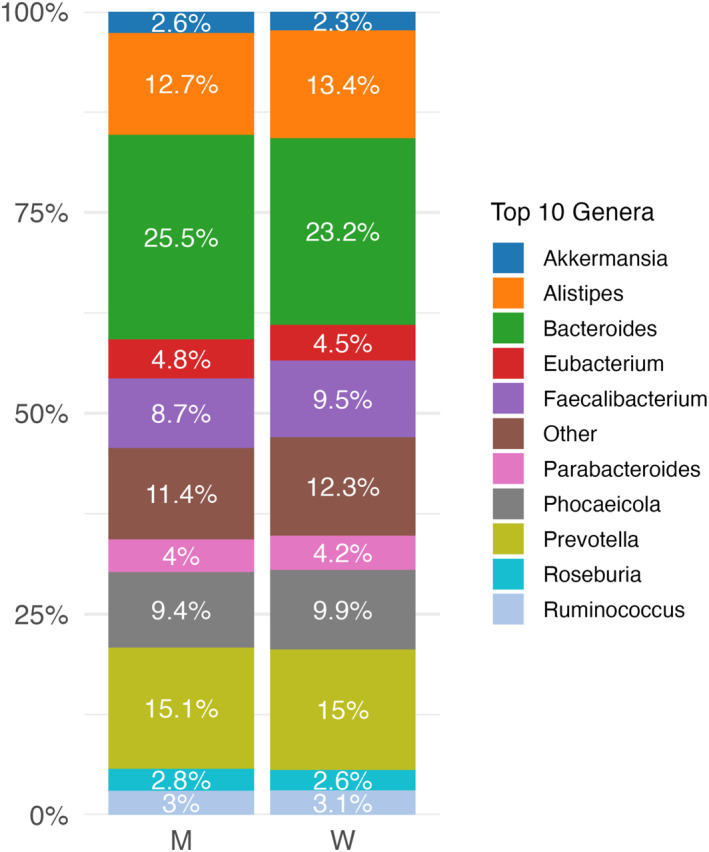
Stacked barplot of the relative abundance (%) of the top 10 most abundant gut bacteria genera in men (M, *n* = 157) and women (W, *n* = 241).

First, we characterised the gut microbiota composition in this sample of women and men at the genus level, including only bacterial genera with a relative abundance greater than 1% (Figure 1). *Bacteroides*, *Prevotella* and *Alistipes* were the three most abundant genera in both men and women (Figure 1). Statistical analysis revealed no significant sex differences in the relative abundance of these genera. Corresponding data are listed in Table 1.

**TABLE 1:** Bacterial genera with a relative abundance (%) greater than 1% in this sample of women and men.

	Women (*n* = 241)		Men (*n* = 157)			
*Gut microbiota genera*	Mean	SD	Mean	SD	*p*‐value	Cohen *d*
*Akkermansia*	1.94%	4.05%	2.28%	4.44%	0.5	0.08
*Alistipes*	11.25%	7.40%	10.90%	9.17%	0.7	−0.04
*Bacteroides*	19.27%	12.97%	22.13%	14.58%	0.05	0.21
*Barnesiella*	1.55%	2.12%	1.44%	1.50%	0.5	−0.06
*Blautia*	1.42%	1.12%	1.28%	1.05%	0.2	−0.12
*Dialister*	1.36%	2.38%	1.23%	1.94%	0.6	−0.06
*Dysosmobacter*	1.22%	0.68%	1.24%	0.76%	0.7	0.03
*Eubacterium*	3.76%	4.12%	4.18%	4.47%	0.3	0.1
*Faecalibacterium*	8.06%	5.67%	7.53%	4.97%	0.3	−0.1
*Flavonifractor*	1.12%	0.70%	1.07%	0.69%	0.5	−0.08
*Gemmiger*	1.35%	1.35%	1.23%	1.14%	0.3	−0.1
*Lachnospira*	1.45%	1.57%	1.61%	2.04%	0.4	0.09
*Parabacteroides*	3.61%	2.48%	3.44%	2.27%	0.5	−0.07
*Phocaeicola*	8.51%	6.64%	8.12%	5.83%	0.5	−0.06
*Prevotella*	12.96%	20.71%	12.53%	21.00%	0.8	−0.02
*Roseburia*	2.16%	1.71%	2.40%	2.44%	0.3	0.12
*Ruminococcus*	2.61%	2.41%	2.60%	2.35%	1	−0.01
*Sutterella*	0.88%	1.37%	0.75%	1.14%	0.3	−0.11

**FIGURE 2:**
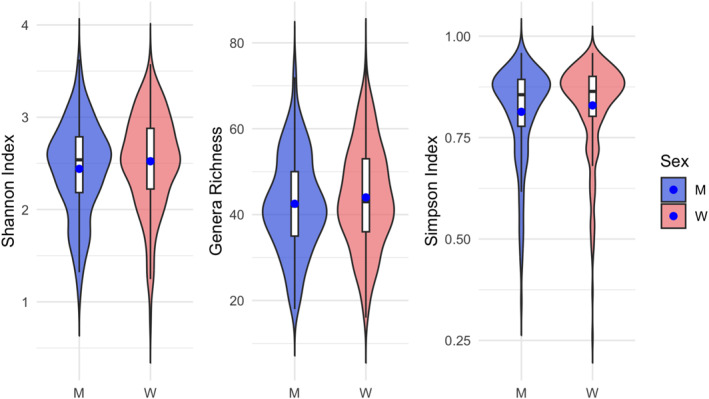
Violin plots for alpha‐diversity indices between men (M, *n* = 157) and women (W, *n* = 241). The blue dot represents the mean, while the horizontal black line represents the median.

To further investigate potential sex differences in gut microbiota composition, we assessed alpha diversity, using the Shannon and Simpson indices, as well as Genera Richness. No significant differences were observed between women and men for any of these indices (Shannon Index, *t* = 2, df = 350, *p* = 0.1; Genera Richness, *t* = 1, df = 356, *p* = 0.2; Simpson Index, Wilcoxon rank‐sum test, *W* = 22,307, *p* = 0.1) (Figure 2).

**FIGURE 3:**
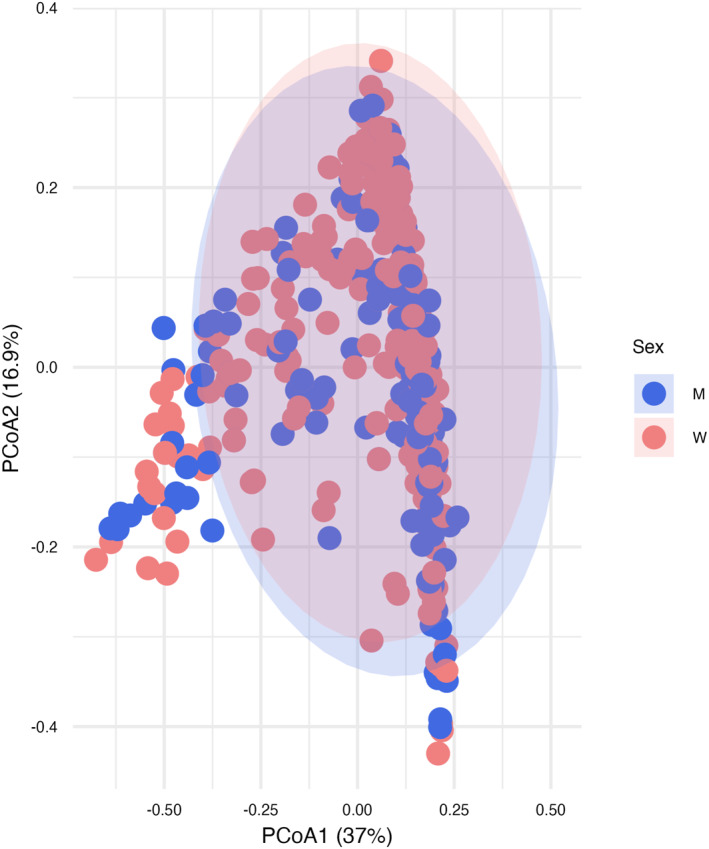
Principal Coordinate Analysis (PCoA) of gut microbiota overall composition (beta‐diversity) in men (M, *n* = 157) and women (W, *n* = 241).

To assess overall gut microbiota composition similarity or difference between men and women, beta diversity was assessed using PCoA, with the two PCoA scores (PC1 and PC2) explaining 53.3% of the variance in the difference distance matrix. No significant sex differences were observed (PERMANOVA *p* = 0.49) (Figure 3).

### 3.3 Perceived Stress and Depressive Symptoms

**FIGURE 4:**
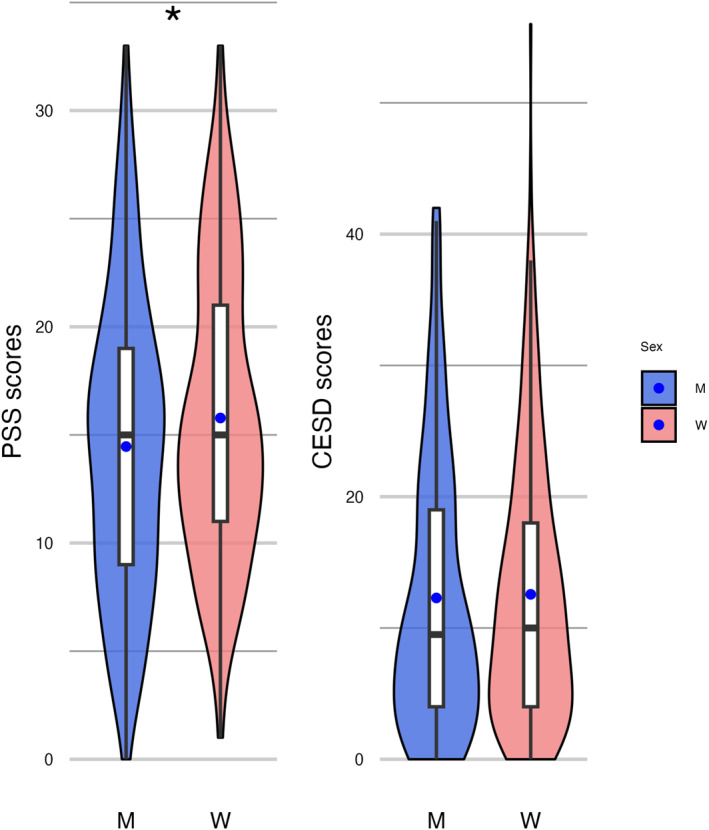
Violin plots for Perceived Stress Scale (PSS) and Centre of Epidemiological Studies Depression Scale (CES‐D) scores in men (M, *n* = 157) and women (W, *n* = 241). The blue dots represent the mean, while the horizontal black lines represent the median.

Perceived stress and depressive symptoms were assessed among participants using the PSS and CES‐D scales, respectively. Two‐hundred participants (122 women) reported a PSS score between 14 and 26, and 42 participants (25 women) reported a PSS score > 26, indicating moderate and high perceived stress, respectively, according to standard cut‐offs. There were no sex differences in the distribution of participants in these categories (chi‐square statistic = 0.04, *p* = 0.98). Also, 60 participants (34 women) reported a CES‐D score between 16 and 23, and 60 participants (35 women) reported a CES‐D score > 23, indicating mild and clinically significant depression, respectively, according to standard cut‐offs. There were no sex differences in the distribution of participants in these categories (chi‐square statistic = 0.70, *p* = 0.70). As shown in Figure 4, there was a marginally significant difference between women and men in mean PSS scores, with women reporting higher scores (*t* = 2, *p* = 0.059, Cohen's d = 0.2). No differences were found between men and women for mean CES‐D scores (*t* = 0.3, *p* = 0.8, Cohen's d = 0.03).

### 3.4 Bayesian Model Averaging

Associations between PSS scores, gut bacteria genera, and their two‐way interaction, with CES‐D scores were modelled using a Bayesian approach.

**FIGURE 5:**
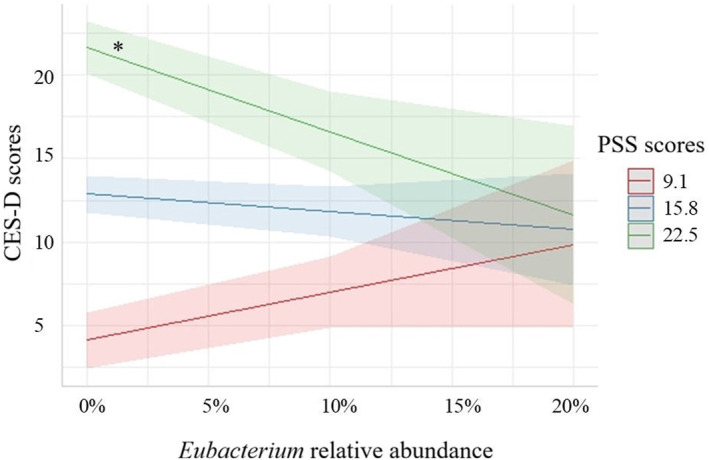
Bayesian prediction model for the effect of the *Eubacterium* genus (relative abundance) on the prediction of depressive symptoms (CES‐D scores) at low, mean, and high perceived stress levels (i.e., PSS scores; higher and lower estimates of PSS scores were derived from ± 1SD from the mean) in women. CES‐D = Centre of Epidemiological Studies Depression Scale; PSS = perceived stress scale. * = 95% CI [−88.77, −11.6], *p* < 0.05.

In women, only PSS scores and the interaction between PSS scores and the relative abundance of a specific gut microbiota genus (*Eubacterium*) showed marginal posterior inclusion probabilities (pip) > 0.5 (PSS: pip = 0.99; PSS×*Eubacterium*: pip = 0.95). Therefore, we focused on these statistical predictors in the subsequent analyses. Specifically, both PSS scores and PSS×*Eubacterium* statistically predicted CES‐D scores in the three top models (i.e., the models with highest Bayes' factors; see Table 2 for further details on the estimated models). Considering the top three models, the estimated coefficients and standard deviations under Bayesian model averaging were obtained (Table 2). The variability of the parameter estimates and associated inferences were computed via 95% Bayesian credible intervals (95% BCIs), which showed higher levels of perceived stress (PSS scores) to be associated with more severe depressive symptoms (CES‐D scores) (*β* = 0.74, 95% BCI = [0.65, 0.82]). Moreover, we also found that *Eubacterium* relative abundance moderated the relationship between psychological stress and depressive symptoms (*β* = −0.16, 95% BCI = [‐0.26; −0.07]). Specifically, post‐hoc slope analysis showed that for women who have higher levels of psychological stress (PSS score), a lower relative abundance of *Eubacterium* is related to higher symptoms of depression (CES‐D score) (Figure 5).

**TABLE 2:** Bayesian analyses for assessing the existence of an association between perceived stress (PSS scores), gut bacteria genera, and their interactions, with depressive symptoms (CES‐D scores) in women and men separately. For each model, the predictors are indicated as either “1” (inclusion of the predictor) or “0” (exclusion of the predictor. Pip: marginal posterior inclusion probabilities; Post *β* (mean ± SD) mean and standard deviation for each coefficient.

	a. Women					b. Men				
			Models					Models		
	Pip	Post *β*	I	II	III	Pip	Post *β*	I	II	III
PSS	0.99	0.74 ± 0.04	1	1	1	0.99	0.70 ± 0.06	1	1	1
*Akkermansia*	0.11	0	0	0	0	0.15	0	0	0	0
*Alistipes*	0.14	0	0	0	0	0.55	0	0	0	0
*Bacteroides*	0.11	0	0	0	0	0.22	0	0	0	0
*Barnesiella*	0.12	0	0	0	0	0.35	0	0	0	0
*Blautia*	0.15	0	0	0	0	0.16	0	0	0	0
*Dialister*	0.14	0	0	0	0	0.22	0	0	0	0
*Dysosmobacter*	0.12	0	0	0	0	0.23	0	0	0	0
*Eubacterium*	0.17	0	0	0	0	0.20	0	0	0	0
*Faecalibacterium*	0.41	−0.03 ± 0.04	0	1	0	0.18	0	0	0	0
*Flavonifractor*	0.11	0	0	0	0	0.73	−0.03 ± 0.06	0	0	1
*Gemmiger*	0.22	0	0	0	0	0.21	0	0	0	0
*Lachnospira*	0.26	0	0	0	0	0.35	0	0	0	0
*Parabacteroides*	0.11	0	0	0	0	0.16	0	0	0	0
*Phocaeicola*	0.16	0	0	0	0	0.19	0	0	0	0
*Prevotella*	0.12	0	0	0	0	0.30	0	0	0	0
*Roseburia*	0.12	0	0	0	0	0.15	0	0	0	0
*Ruminococcus*	0.12	0	0	0	0	0.17	0	0	0	0
*Sutterella*	0.12	0	0	0	0	0.17	0	0	0	0
PSS×*Akkermansia*	0.12	0	0	0	0	0.15	0	0	0	0
PSS×*Alistipes*	0.15	0	0	0	0	0.50	0	0	0	0
PSS×*Bacteroides*	0.29	0	0	0	0	0.16	0	0	0	0
PSS×*Barnesiella*	0.36	−0.01 ± 0.03	0	0	1	0.16	0	0	0	0
PSS×*Blautia*	0.12	0	0	0	0	0.42	0.03 ± 0.06	0	1	0
PSS×*Dialister*	0.16	0	0	0	0	0.16	0	0	0	0
PSS×*Dysosmobacter*	0.12	0	0	0	0	0.16	0	0	0	0
PSS×*Eubacterium*	0.94	−0.16 ± 0.05	1	1	1	0.21	0	0	0	0
PSS×*Faecalibacterium*	0.22	0	0	0	0	0.16	0	0	0	0
PSS×*Flavonifractor*	0.12	0	0	0	0	0.16	0	0	0	0
PSS×*Gemmiger*	0.12	0	0	0	0	0.29	0	0	0	0
PSS×*Lachnospira*	0.29	0	0	0	0	0.16	0	0	0	0
PSS×*Parabacteroides*	0.13	0	0	0	0	0.23	0	0	0	0
PSS×*Phocaeicola*	0.14	0	0	0	0	0.18	0	0	0	0
PSS×*Prevotella*	0.19	0	0	0	0	0.18	0	0	0	0
PSS×*Roseburia*	0.20	0	0	0	0	0.41	0	0	0	0
PSS×*Ruminococcus*	0.32	0	0	0	0	0.17	0	0	0	0
PSS×*Sutterella*	0.18	0	0	0	0	0.28	0	0	0	0
Bayes factor			1	0.49	0.33			1	0.57	0.53
*R* ^2^			0.57	0.58	0.58			0.50	0.51	0.51

In men, Bayesian analysis revealed that PSS scores and two gut bacteria genera (i.e., *Alistipes* and *Flavonifractor*) showed pip > 0.5 (PSS: pip = 0.99; *Alistipes*: pip = 0.55; *Flavonifractor*: pip = 0.73). Considering the top three models, higher levels of perceived stress (PSS score) were associated with more severe depressive symptoms (CES‐D score) in men (*β* = 0.70, 95% BCI = [0.59; 0.81]). Conversely, there was no evidence for associations between gut bacteria genera and CES‐D scores, nor PSS x gut bacteria genera interactions with CES‐D scores in men (see Table 2).

## 4 Discussion

To our knowledge, this is the first study to assess whether perceived stress and the relative abundance of specific gut bacteria genera are associated, independently and/or in combination, with the severity of depressive symptoms in healthy adults in a sex‐specific manner.

Using a Bayesian approach, we observed that perceived stress was an independent predictor of depressive symptoms in both men and women (Kendler et al. 1999). Crucially, in women, the genus *Eubacterium* moderated this relationship. In fact, higher perceived stress combined with lower *Eubacterium* relative abundance predicted more severe depressive symptoms. On the other hand, gut bacteria genera did not moderate the relationship between perceived stress levels and depressive symptoms in men.

The genus *Eubacterium* is part of the core human gut microbiota, consistently colonising the human gut across various populations. Some *Eubacterium* species are known producers of SCFAs, especially butyrate and propionate, which contribute to maintaining intestinal barrier stability (Dalile et al. 2019) and whose levels are reduced following acute stress (Rosell‐Cardona et al. 2025). Furthermore, SCFAs modulate the immune response through inhibition of histone deacetylase activity or interaction with free fatty acids receptors expressed on immune cells, leading to a reduced production of pro‐inflammatory cytokines (Lin et al. 2024; Mukherjee et al. 2020). SCFAs can also directly activate vagal afferent terminals (Lal et al. 2001), thereby modulating vagal activity and signalling to the brain, which has also been shown to exert anti‐inflammatory properties (Tracey 2007). Therefore, we speculate that a lower abundance of *Eubacterium* genera might be associated with a smaller production of SCFAs, leading to greater levels of inflammation (Mukherjee et al. 2020) which could contribute to the pathophysiology of depression (Lee et al. 2025; Lin et al. 2024).

Supporting our results, a reduced abundance of the genus *Eubacterium* has been reported in the gut of patients with major depressive disorder compared to healthy controls (Yang et al. 2020), and particularly among those with more severe depressive symptoms (Hu et al. 2023). A recent study also reported a lower abundance of the genus *Eubacterium* in patients with irritable bowel syndrome, particularly in those exhibiting comorbid psychological symptoms such as anxiety and depression (Liu et al. 2025). Moreover, a human study found a positive correlation between *Eubacterium* abundance and positive mood changes, while reductions in its abundance were associated with worsening negative mood, suggesting that the genus *Eubacterium* may represent a potential psychobiotic candidate (Hao et al. 2023). Furthermore, a recent study showed that in healthy individuals with lower cardiac vagal modulation, a reduced abundance of the genus *Eubacterium* in the gut was associated with greater depressive symptoms (Ravenda et al. 2025). Preclinical studies further support a potential role for the genus *Eubacterium* in mood modulation, as increased *Eubacterium* abundance in the gut was positively associated with prosocial behaviour in rats (Hazani et al. 2025), and inoculation with *Eubacterium rectale* reduced anxiety and depressive‐like behaviours in chronic stress animal models (Hao et al. 2023).

Besides *Eubacterium*, several other SCFA‐producing bacteria genera have been found to be reduced in patients with depression, such as *Faecalibacterium*, *Coprococcus*, *Ruminococcus*, *Bifidobacterium*, *Dialister*, *Blautia* (X. Chen et al. 2024; Sanada et al. 2020; Yang et al. 2020). Notably, the *Faecalibacterium* genus includes *Faecalibacterium prausnitzii* that has been shown to improve depressive and anxiety‐like behaviours in animal models (Hao et al. 2019). As such, *Faecalibacterium prausnitzii*, *Eubacterium rectale,* and other SCFA‐producing bacteria species have been proposed as potential psychobiotics with beneficial effects on mood (Hao et al. 2023).

In this sample of healthy adults, no sex differences were found in terms of gut microbiota composition. With regard to sex differences in psychometric characteristics, women reported higher perceived stress (small effect size) than men, whereas no differences were found in depressive symptom severity. Previous research in patients with mood and gastrointestinal disorders has shown sex‐specific patterns in indices of gut microbiota diversity (J. Chen et al. 2018; Manosso et al. 2021). For example, female patients with depression exhibit greater diversity compared to male patients (Y. S. Kim et al. 2020; Y. Li et al. 2022). However, a study conducted on a healthy sample of the Japanese population found no sex differences in alpha diversity, but beta diversity analysis revealed distinct gut microbial structures between sexes (Takagi et al. 2019). This study also reported sex differences in the relative abundances of major gut microbiota genera (Takagi et al. 2019). Two other studies on different healthy populations revealed sex differences in overall gut microbiota composition and relative abundances at the phylum level (Dominianni et al. 2015; Koliada et al. 2021). Another study reported sex‐related differences in beta diversity in mucosa‐associated microbiota, which is less influenced by diet than stool‐associated microbiota (Borgo et al. 2018), suggesting that while diet is a major factor influencing gut microbiota composition and function, certain gut microbial niches may be more strongly shaped by sex than others.

Converging evidence suggests that sex hormones may contribute to sex‐specific vulnerability to depressive symptoms through their modulation of stress reactivity and gut microbiota composition. In females, fluctuations in oestrogen levels across the menstrual cycle and lifespan have been linked to stress and mood regulation, with periods of high oestrogen and low progesterone associated with increased hypothalamic–pituitary–adrenal (HPA) axis reactivity (Viau and Meaney 1991) and the reproductive years marked by greater vulnerability to depression and other mood disturbances (Barth et al. 2015; Luo et al. 2024). In contrast, testosterone seems to exert a buffering effect on HPA axis reactivity to stress in animal models (Viau and Meaney 1996) and on depressive symptoms in men (Pope et al. 2003). Moreover, sex hormones strongly influence gut microbiota composition with sex differences emerging after puberty (Eltokhi and Sommer 2022; Y. S. Kim et al. 2020; Santos‐Marcos et al. 2023). In animal models, sex differences in the composition of gut microbiota disappear after gonadectomy and are restored following treatment with sex hormones (Org et al. 2016; Organski et al. 2025). As such, sex differences in gut microbiota composition may further contribute to sex‐specific immune activation and microbial metabolic pathways, which could contribute to sex disparities in depression risk (Chang et al. 2024; Lu et al. 2017; Rosell‐Cardona et al. 2025). While evidence from preclinical and clinical settings is robust (J. Chen et al. 2018; Manosso et al. 2021), findings on healthy human populations remain heterogenous (Borgo et al. 2018; Dominianni et al. 2015; Koliada et al. 2021; Takagi et al. 2019), warranting further research to clarify sex‐specific gut microbiota‐brain interactions.

### 4.1 Limitations

We acknowledge that great caution must be taken in interpreting the current results, given the following limitations. First, we adopted a Bayesian approach to test associations between stress, gut microbiota genera and depressive symptoms, but the cross‐sectional nature of this exploratory study limits the interpretation of the results in terms of cause‐effect relationships. Second, data on participants' dietary habits (including the use of probiotics) and lifestyle, both of which can influence gut microbiota composition (Conlon and Bird 2014; Pandit et al. 2025), were not available. Likewise, we do not have any information regarding the menstrual cycle phase or the use of contraceptives among female participants, which could potentially impact mood regulation and gut microbiota composition (K. L. Chen and Madak‐Erdogan 2016; Leao et al. 2025). Also, we were underpowered to study sex differences in different age groups. Third, gut microbiota analysis was conducted at genus‐level, limiting the possibility to infer microbial function because of significant functional diversity within genera. Lastly, participants were recruited within a small urban area, therefore generalisability should be tested in diverse populations and settings.

### 4.2 Conclusion

In conclusion, the results of this study support clinical evidence on the relationship between the genus *Eubacterium* and depressive symptoms. Importantly, the current study extends previous research by showing that, in healthy women, higher perceived stress combined with lower *Eubacterium* abundance predicted more severe depressive symptoms. Notwithstanding the above reported limitations, the current results (i) warrant further investigation of the interactions among stress, specific gut microbiota features, and depressive symptoms in non‐clinical populations, (ii) highlight the importance of considering sex as a key biological variable in these interactions, and (iii) suggest that the *Eubacterium* genus is a promising microbial biomarker associated with depression in women reporting higher stress levels. Future studies with species‐level resolution and longitudinal designs are needed to determine the extent to which changes in perceived stress and depressive symptoms are associated with changes in the abundance of specific *Eubacterium* species in the gut, and whether this is consistent with the speculated SCFA‐related mechanisms. Also, it would be interesting to test whether probiotic supplementation targeting *Eubacterium*‐related pathways ameliorates depressive symptoms, particularly in women under higher stress levels.

## Conflicts of Interest

The authors declare no conflicts of interest.

## Data Availability

The data that support the findings of this study are available from the corresponding author upon reasonable request.
